# Adverse childhood experiences and health-related quality of life in adulthood: revelations from a community needs assessment

**DOI:** 10.1186/s12955-015-0323-4

**Published:** 2015-08-11

**Authors:** Abraham A. Salinas-Miranda, Jason L. Salemi, Lindsey M. King, Julie A. Baldwin, Estrellita “Lo” Berry, Deborah A. Austin, Kenneth Scarborough, Kiara K. Spooner, Roger J. Zoorob, Hamisu M. Salihu

**Affiliations:** Department of Epidemiology and Biostatistics, College of Public Health, University of South Florida, 13201 Bruce B Downs, MDC56, Tampa, Florida 33612 USA; REACHUP Inc., 2902 N, Armenia Avenue Suite 100, Tampa, Florida 33607 USA; Department of Family and Community Medicine, Baylor College of Medicine, 3701 Kirby Drive, Suite 600, Houston, Texas 77098 USA; Department of Community and Family Health, College of Public Health, University of South Florida, 13201 Bruce B. Downs Blvd. MDC 56, Tampa, Florida 33612 USA

## Abstract

**Background:**

Adverse childhood experiences (ACE) have been previously linked to quality of life, health conditions, and life expectancy in adulthood. Less is known about the potential mechanisms which mediate these associations. This study examined how ACE influences adult health-related quality of life (HRQoL) in a low-income community in Florida.

**Methods:**

A community-based participatory needs assessment was conducted from November 2013 to March 2014 with 201 residents of Tampa, Florida, USA. HRQoL was measured by an excessive number of unhealthy days experienced during the previous 30-day window. Mediation analyses for dichotomous outcomes were conducted with logistic regression. Bootstrapped confidence intervals were generated for both total and specific indirect effects.

**Results:**

Most participants reported ‘good to excellent health’ (76 %) and about a fourth reported ‘fair to poor health’ (24 %). The mean of total unhealthy days was 9 days per month (SD ±10.5). Controlling for demographic and neighborhood covariates, excessive unhealthy days was associated with ACE (AOR = 1.23; 95 % CI: 1.06, 1.43), perceived stress (AOR = 1.07; 95 % CI: 1.03, 1.10), and sleep disturbance (AOR = 8.86; 3.61, 21.77). Mediated effects were significant for stress (β = 0.08) and sleep disturbances (β = 0.11) as they related to  the relationship between ACE and excessive unhealthy days.

**Conclusion:**

ACE is linked to adult HRQoL. Stress and sleep disturbances may represent later consequences of childhood adversity that modulate adult quality of life.

**Electronic supplementary material:**

The online version of this article (doi:10.1186/s12955-015-0323-4) contains supplementary material, which is available to authorized users.

## Background

Racial/ethnic minorities in the United States suffer a disproportionately high burden of morbidity and mortality compared to their non-Hispanic white counterparts [1], particularly with respect to adverse pregnancy outcomes, childhood illnesses, and adult chronic diseases [1]. These health differences are likely the product of complex relationships across social, economic, environmental, healthcare, bio-behavioral, structural factors such as discrimination and racism, as well as literacy and legislative policies [2]. In addition to stressors experienced during adulthood, stressors and adverse experiences during childhood can also have a long-term impact on health [3], and consequently, quality of life.

According to the Centers for Disease Control and Prevention (CDC), more than half of adults in the U.S. have suffered from adverse childhood experiences (ACE), such as verbal, physical abuse and family dysfunction [4]. These exposures have been linked to a variety of health conditions in adults, including depression, cardiovascular disease, cancer, diabetes [5, 6], as well as premature mortality [7]. Moreover, research suggests that ACE is associated with long-term changes in the nervous, endocrine, and immune systems [3, 8]. This damage to the body’s stress response system, coupled with the adoption of poor health behaviors to help cope with stress [9], appears to contribute to a deterioration of adult health. Indeed, the enduring impact of ACE on health suggests that the overall health-related quality of life (HRQoL) for these adults may also be impacted [10]. Although evidence exists linking adversity during the childhood years (i.e., adverse childhood experiences or ACE) to impaired HRQoL and shortened life expectancy in adulthood [7, 11–15], little is known about the potential mechanisms that mediate the relationship. Further, there have been even fewer empirical studies conducted in socioeconomically disadvantaged populations assessing a wide range of interconnected risk and protective factors that influence the HRQoL of individuals within a community context. To improve the health and quality of life of minority populations, an enhanced understanding of the factors that contribute to health disparities is needed. Accordingly, we conducted this study to examine how ACE influences adult-onset HRQoL in a community setting. We also explored the roles played by key mediating socio-demographic and other neighborhood level factors and assessed plausible mediating pathways.

## Methods

### Context

The project *“Toward Eliminating Disparities in Maternal and Child Health Populations”* is a 3-year CBPR (community-based participatory research) initiative that partners researchers at the University of South Florida (USF), a non-profit advocacy and empowerment organization, REACHUP, Inc., and a group of community representatives from selected zip codes in Central Tampa, Hillsborough County, Florida. The project has three sequential phases: 1) needs assessment to identify priority maternal and child health issues, 2) planning an evidence-based intervention that addresses the priorities identified, and 3) implementation of a pilot community-driven intervention. The project is funded by the National Institute on Minority Health and Health Disparities (5R24MD8056-02).

This paper focuses exclusively on the needs assessment survey, which was designed to explore health determinants and quality of life indicators in the target community, implemented using a CBPR approach. This CBPR study builds extensively upon a strong, existing community-academic partnership. At the study’s outset, a Community Advisory Board (CAB) was created, which consisted of eight adult residents from the target community who were recruited based upon their knowledge, participation, advocacy, and leadership in previous community projects [16, 17]. In addition to the required Human Subjects Protection Certification course, all CAB members completed skill-building workshops in research methods. Active participation of community members occurred in the design, data collection, and analysis phases of the study.

### Design

The *community needs assessment survey* was a cross-sectional instrument designed to assess factors associated with HRQoL and was administered between November 2013 and March 2014. Two-hundred and one adult participants were recruited from approximately 110,451 residents of the target population [18]. The target community was predominantly African American (60 % black, 18.3 % white, 12.1 % Hispanic, and 9.6 % other) and tended to be economically disadvantaged, with half the income and double the unemployment rate of the rest of the county [19, 20]. The survey was administered through intercept interviews across a five zip code area [21, 22]. *Intercept interviews* are a type of social marketing research in which respondents are approached by culturally and linguistically matched research staff and “intercepted” in various places where community members gather often [23]. This method is used to collect more representative information than convenience samples regarding particular issues of concern to the community, and intercept interviews are more feasible and less expensive than door-to-door needs assessments. Intercept interviews are also useful when the target population is widely dispersed and harder to reach (e.g., several zip code-level areas with economically disadvantaged urban communities). Accordingly, trained CAB members surveyed people “in the streets” at frequently used community locations (e.g., cafes, churches, libraries, local schools gatherings, shopping centers) and at times when community residents were accessible (e.g., weekends, after hours during weekdays). In a similar manner to 30 by 7 cluster sampling [24], the CAB nominated 30 community locations or “clusters” (23 were actually surveyed due to redundancy of participants in 7 locations) based on socio-demographic and economic characteristics using zip code level census data, and then randomly sampled at least seven individuals from each location. To participate in the survey, a respondent must have been a community resident for at least the previous 12 months. Flyers, social media, and “word-of-mouth” transmission of information were used for recruitment. Written informed consent was obtained and a modest but appropriate monetary incentive was given to study participants. Approval for the study was obtained from the Institutional Review Board of the University of South Florida.

### Measures

The development of survey questions was guided by the Life Course Perspective (LCP) with extensive input from the CAB. The LCP framework is used to evaluate the cumulative influences of risk and protective factors during critical periods of human development and the effects those events have on the health trajectories of individuals [25–27]. First, community members, in partnership with academic researchers, reviewed local data and voiced their concerns for critical topics. Next, academic researchers suggested questionnaires or scales that had been validated in previous research studies for local adaptation. Subsequently, the CAB provided feedback on wording and readability, question addition/deletion, assessed acceptability of questions and technology usability (see Technology section), and pilot tested questions before community-wide implementation. The final survey questionnaire contained 63 questions (Likert-type and multiple choice questions), which covered the following inquiry domains and specific sequencing into the survey: life in the neighborhood (neighborhood assets; community-wide issues), social connections (social support), health and quality of life (general health, HRQoL, self-reported health problems, sleepless days, perceived social standing), stress and unfair treatment (stress appraisal, perceived experiences of discrimination), lifestyle (smoking, alcohol use, recreational drugs, diet, exercise, perceived HIV risk), childhood experiences (ACE), and socio-demographic questions (age, education, marital status, race/ethnicity, household income, employment). Academic researchers assisted with the development of hypotheses, analytic strategies, and statistical analyses. In the subsections below, we describe only the specific instruments and measures used to test our main hypotheses; however, the complete survey is available as a supplemental file (Additional file 1).

### Primary outcome: health-related quality of life

HRQoL was the primary study outcome and was measured using the CDC’s “Healthy Days Measure” instrument, which is a validated scale used frequently in national health surveillance surveys [28, 29]. Specifically, we used the brief version, referred to as Healthy Days Core Module (4-items questionnaire), which captures the self-reported number of days in the past 30 days that individuals rated their physical or mental health as not good [30]. It includes the following components: 1) self-rated health, from poor to excellent (ordinal); 2) number of days when physical health was not good during the past 30 days; 3) number of days when mental health was not good during the past 30 days; and 4) number of activity limitations due to either physical or mental health illness (combined). Total number of unhealthy days is then obtained by adding the responses to questions 2 and 3. Any sum greater than 30 was capped at 30, with a maximum of 30 unhealthy days [30]. The outcome was operationalized through dichotomization of the total number of unhealthy days, with poor HRQoL reflected by 14 or more total days. This cut-point has been used by other authors as a discriminate measure to assess excessive unhealthiness [31–33].

### Primary exposure: adverse childhood experiences

To assess cumulative risk from childhood to adulthood, the main predictors included participant recall of adverse events that occurred during the first 18 years of life. We used the Brief Family History Questionnaire from the ACE study [6], a 10-item questionnaire collecting self-reported physical, emotional, and sexual abuse, family dysfunction, and economic hardship. All items are dichotomous, yes/no questions. An overall ACE score, which ranges from 1–10, is then calculated by adding the number of “yes” responses. Higher scores have been found to be associated with a wide range of adverse health outcomes, impaired quality of life, and higher mortality [7, 11, 34].

### Potential mediators: smoking, alcohol use, diet, physical activity, and sleep disturbances

We hypothesized that contemporary lifestyle risk and protective factors of participants could mediate the association between ACE and adult HRQoL. Lifestyle and behavioral questions were obtained from multiple validated instruments that are frequently used in health services research, which were adapted using input from community members. *Smoking* was assessed with a question from the 2013 Behavioral Risk Factor Surveillance System (BRFSS) [35]: “Have you smoked at least 100 cigarettes in your entire life?” This question was chosen by CAB members as being easier to respond to than a detailed frequency question [36]. The frequency of *alcohol use* per month was measured with a numerical question, also adapted from the 2013 BRFSS: “In a typical month, how often do you drink alcoholic beverages?”

Although self-reported measures of dietary patterns are vulnerable to measurement bias [37, 38], the CAB members felt that asking a few questions about diet was still an important aspect to include in the survey as a general, non-sensitive behavioral item. Thus, the survey included a short series of questions about intake of fruits and vegetables, fatty, sugary, salty foods, and caffeinated drinks. Participants were asked to indicate how often they consumed fruits and vegetables in a typical month, and we grouped responses as: “once a month or less” coded as 1, “2–3 times a month” coded as 2.5, “once a week” coded as 4, “2–3 times a week” equivalent to 10, and “4–6 times a week or more” coded as 20. For this study, we focused on the *consumption of fruits and vegetables* as key protective factor because of stronger evidence regarding fruits and vegetable self-reported measures. Specifically, brief instruments for fruit and vegetable intake assessments have been found to be adequate for estimating relative risks in the relationship between fruit and vegetable intake versus disease [39]. Because the survey included questions not previously validated, we consider our approach a conservative one. Moreover, because we did not find that any of our self-reported dietary measures were significantly associated with HRQoL in bivariate preliminary analyses, we did not explore dietary items in greater detail in mediational analyses. *Sleep disturbances* were also measured with a question we derived from the BRFSS [28]: “During the past 30 days, for about how many days have you felt you did not get enough rest or sleep?” *Physical activity* was measured with one question that assessed physical activity, also from the BRFSS [28]: “During the past month, about how many days per week did you exercise for recreation or to keep in shape (activities that make you sweat)?”

### Stress appraisal

Cognitive appraisal of stress was measured with the 4-item Perceived Stress Scale, which is a validated instrument used to make comparisons of subjects’ perceived stress related to current events [40]. Questions are 5-point Likert type scaled (i.e., strongly disagree = 1, to strongly agree = 5). A composite score was derived by adding the original scores and multiplying by a factor of 5, which results in a 100-point scale. The higher the score, the higher the risk for clinical psychiatric disorder [41].

### Social support

Perceived social support was measured with five questions from the Medical Outcomes Study Social Support Survey [42]. Using 5-point Likert type scales (‘None of the time’ = 1 to ‘All of the time’ = 5), individuals were asked to indicate how often the following types of supports were available to them if needed: 1) someone to confide in or talk about yourself or your problems; 2) someone to share your most private worries and fears with; 3) someone to help you if you were confined to bed; 4) someone to prepare your meals if you were unable to do it yourself; and 5) someone to get together with for relaxation. Question scores were added to yield a total social support score. To facilitate interpretation in the community setting, the original scores ranging from 4 to 20 were converted to a 100-point scale by multiplying the original score by five.

### Sociodemographic confounders

Socio-demographic characteristics were collected from participants and included: age in years (categorized as 18–35; 36–45; 46–55; and ≥ 56 years), sex, education (high school vs. less than high school graduate or equivalent), current marital status, race/ethnicity (non-Hispanic white, non-Hispanic black, Hispanic, other), employment status (employed, unemployed but able, and unable to work), annual household income (categories: US$ 0–20,000; 20,001–40,000; and ≥ 40,000), and residential time in the five target zip codes (5 years or less vs. more than 5 years).

### Neighborhood confounders

Community resources, community-wide issues, and neighborhood cohesion levels were considered as potential confounders in our analyses. First, resources available in the community were measured with the following question: “Which of the following resources are available to you in your community?” A list of resources was compiled using CAB input and provided with the survey. Each item listed was weighted equally and the final variable used in analyses was the total number of assets or resources reported. Similarly, perceived community-wide issues were measured with one question on neighborhood problems [43]: “Which of the following is a problem in the neighborhood?” Again, a list was developed by the CAB, with the allowance for write-in entries. The final variable for analysis consisted of the total number of different issues reported. Lastly, neighborhood social cohesion was assessed by measuring the respondent’s level of agreement (on a 5-point Likert type scale) with a set of questions proposed by Cagney and colleagues [44]: 1) “People around here are willing to help their neighbors”; 2) “This is a close-knit neighborhood”; 3) “People in this neighborhood can be trusted”; and 4) “People in this neighborhood generally don’t get along with each other” (reverse coded). These questions were then summed to provide a total score, where higher scores indicated higher neighborhood social cohesion.

### Technology

The droidSURVEY software was used to design and administer the survey [45], which was installed on ten Hewlett-Packard Slate 7” portable tablet computers running the Android™ 4.2.2 operating system [46]. For Spanish-speaking participants, all questions were translated to Spanish by native speakers and assessed for accuracy of translation using back translation and pilot testing. The use of tablet-administered surveys allowed for a portable, convenient means of data collection, and pilot testing revealed that community members found touchscreen technology to be intuitive, easy to use, and enjoyable. Supervised by the principal investigator, the study coordinator trained community members in the use of tablets and survey implementation, the informed consent process, management of tablet computers in the field, and how to assist participants with questions about the study. Training consisted of three 2-hour sessions in the preceding month to the survey.

### Statistical analysis

The study population was described using descriptive statistics that included frequencies and percentages for categorical variables and means and standard deviations for numerical variables. Two-sided tests of equality for column proportions (*z*-tests for column proportions or *t*-tests for column means) were conducted to assess differences by outcome group. Tests assumed equal variances and were adjusted for multiple comparisons using a Bonferroni correction. All analyses were conducted with the IBM SPSS Statistics for Windows, Version 22.0 (IBM Corp, Armonk, NY). Statistical significance was assessed at the 0.05 level.

Under the LCP framework, we posited that numerous accumulating risks and protective factors mediate the association between ACE and the HRQoL proxy. As a first step, separate logistic regression models were run to assess the independent effects of life course social determinants on the outcome (≥14 unhealthy days), adjusting for individual socio-demographic and neighborhood covariates. We explored the role of the following factors: stress, sleep disturbances, smoking, alcohol use, physical activity, fruits and vegetable intake, and social support. The purpose was to identify significant independent associations with HRQoL that could represent potential mediating pathways in the relationship between ACE and HRQoL.

After a set of factors was identified as possible mediators, the next step was to test for mediation or indirect effects [47]. The following three conditions must be established to determine whether mediation had occurred (i.e., indirect effects) [48]: 1) that the independent variable (ACE) predicts the dependent variable (HRQoL), 2) that the independent variable (ACE) predicts the mediators (Ms), 3) that the mediator (M) predicts the dependent variable (i.e., ≥14 unhealthy days). Additionally, the effects of confounders were considered [49].

Accordingly, mediation analyses for the dichotomous outcome (Y: HRQoL) were conducted with logistic regression models to estimate the path coefficients in a two-mediator model (X: ACE, M1: stress, M2: sleep disturbance). We controlled for socio-demographic covariates (i.e., age, gender, race/ethnicity, no high school, and household income) and neighborhood level factors (i.e., neighborhood cohesion, community resources, and neighborhood issues). The first step was the estimation of controlled direct effects through a series of logistic regressions. This was followed by the decomposition of effects into total, indirect, and direct effects [50]. The formula used to calculate indirect effects for both mediators was: *c = c’ + (ab)*; where *c* is total effect, *c’* is the direct effect, and a*b is the indirect effect. The indirect effect (ab) is the measure of the amount of mediation, and equals the reduction of the effect of the causal variable on the outcome or: ab = c - c’. For this purpose, we used an SPSS macro with asymptotic and resampling strategies for comparing indirect effects in multiple mediator models, which included bootstrapping to estimate confidence intervals for total and specific indirect effects [47, 51]. Missing values were handled with default option in SPSS for logistic regression procedure, which is listwise deletion.

## Results

### Participants’ characteristics, neighborhood factors, and health-related quality of life

Table 1 describes the study sample, by socio-demographic characteristics. In total, 201 participants were surveyed, of which the majority were female (65.7 %) and non-Hispanic black (66.5 %), with a mean age of 45 ± 14 years. Nearly one-fifth of participants did not graduate from high school, most were not married (67 %), and only 40 % were currently employed. Income was not reported by 13 % of participants. Among those who reported income, the majority had incomes lower than $20,000 (about 70 %). Moreover, nearly all participants (97.5 %) reported receiving at least one form of social assistance based on federal poverty line categorizations (i.e., Section 8-Housing Choice Voucher Program, food stamps, school free or reduced lunches, Medicaid, supplemental security income, Temporary Assistance for Needy Families, or other). This was also a relatively transient population, with as many residents who had lived for 5 or more years in the neighborhood as those that had just moved in the last 5 years.

**Table 1:** Unhealthy days during the previous 30 days by participants’ characteristics and neighborhood factors

	Total		0–13 unhealthy days a month		14 or more unhealthy days a month	
	N^b^	(%)	Count	(%)	Count	(%)
Age (in years)						
18–35 years	62	(31.0)	46	(31.3)	16	(30.2)
36–45 years	39	(19.5)	31	(21.1)	8	(15.1)
46–55 years	50	(25.0)	38	(25.9)	12	(22.6)
56 plus years	49	(24.5)	32	(21.8)	17	(32.1)
Sex						
Female	132	(65.7)	93	(62.8)	39	(73.6)
Male	69	(34.3)	55	(37.2)	14	(26.4)
Education						
High school complete	162	(80.6)	124	(83.8)	38	(71.7)
No high school	39	(19.4)	24	(16.2)	15	(28.3)
Marital status						
Currently married^a^	65	(32.7)	54	(37.0)	11	(20.8)
Not married now^a^	134	(67.3)	92	(63.0)	42	(79.2)
Race/ethnicity						
Non-Hispanic whites	13	(6.5)	8	(5.4)	5	(9.4)
Non-Hispanic blacks	133	(66.5)	98	(66.7)	35	(66.0)
Hispanic or Latino	54	(27.0)	41	(27.9)	13	(24.5)
Household annual income						
$0–20,000^a^	114	(56.7)	77	(38.3)	37	(18.4)
$20,001–40,000	36	(18.0)	29	(14.4)	7	(3.5)
$40,001 or more	24	(11.9)	21	(10.4)	3	(1.5)
Income not reported	27	(13.4)	21	(10.4)	6	(3.0)
Residential time						
5 years or less	101	(50.2)	76	(51.4)	25	(47.2)
More than 5 years	100	(49.8)	72	(48.6)	28	(52.8)
Neighborhood factors	Mean	SD	Mean	SD	Mean	SD
Community assets	7.78	(3.5)	7.88	3.38	7.49	3.92
Community wide issues	4.11	(2.9)	4.03	2.93	4.36	2.97
Neighborhood cohesion	65.33	(18.7)	66.03	18.81	63.40	18.73

Column comparisons of 0–13 unhealthy days a month vs. 14 or more unhealthy days. ^a^Values were significantly different at *p* < .05 in the two-sided test of equality for column proportions (z-tests for column proportions or t-tests for column means). ^b^Some column numbers do not add to total sample size due to missing values

**Fig. 1:**
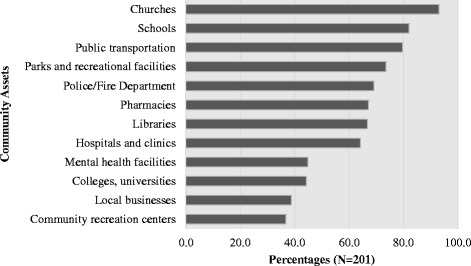
Community assets reported by survey participants. Notes: Percentages based on *N* = 201

Participants demonstrated awareness of community resources, reporting an average of 8 community assets (range was from 0 to 13 reported assets). The most frequently cited resources were churches and schools (Fig. 1). Other high-ranking community resources (in order of highest frequency to least) were public transportation, parks/recreational facilities, police/fire departments, pharmacies, libraries, and hospitals/clinics.

**Fig. 2:**
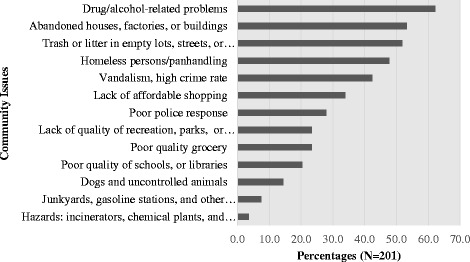
Community-wide issues reported by survey participants. Notes: Percentages based on *N* = 201

Participants also reported community-wide issues that were a perceived problem for their health and their neighborhood (Fig. 2). On average, participants noted about 4 community-wide issues (SD ± 2.9). Drug and alcohol related issues were the most frequently stated followed by abandoned property, litter, homeless issues, high crime rate, lack of affordable shopping, poor police response, poor quality grocery stores, and lack of parks/recreational facilities.

Mean neighborhood cohesion was 65.33 (SD ±18.7), and the majority (60.3 %) of participants perceived that people in their neighborhood generally got along with each other. More than half (57 %) perceived their neighborhood as a place where people were willing to help each other. On the other hand, only 41.5 % of participants agreed or strongly agreed that people in their neighborhood shared the same values. In addition, only 40.4 % of participants agreed or strongly agreed that they lived in a close-knit neighborhood.

**Fig. 3:**
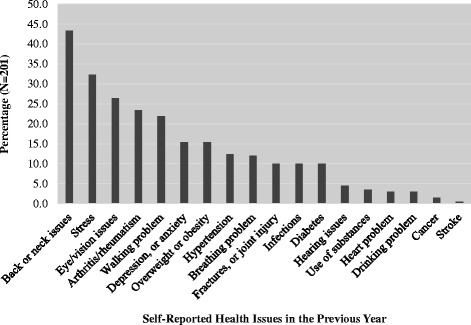
Self-reported health issues among survey participants. Notes: Percentages based on *N* = 201

Approximately three of every four participants considered themselves in “good to excellent health.” The mean number of unhealthy days due to poor physical health or injury in the past month was 4.9 days (SD ± 7.8). The mean number of unhealthy days due to stress, depression or problems with emotions was 5.3 days (SD ± 8.4). When combined both physically and mentally unhealthy days, the mean total unhealthy days was about 9 days a month (SD ± 10.54). About a fourth of participants (26 %) reported having 14 or more total unhealthy days per month (Table 1). The most common self-reported personal health issues included back/neck problems, stress, vision problems, arthritis, problems walking, depression/anxiety, and weight issues (Fig. 3).

Marital status was significantly associated with ≥14 or more unhealthy days (Table 1). Specifically, a higher proportion of unmarried individuals in the group reported ≥14 or more unhealthy days (79.2 %). Also, low income was associated with ≥14 or more unhealthy days, specifically a higher proportion of individuals with incomes lower than $20,000 occurred among those who reported ≥14 or more unhealthy days. We found no significant differences by other socio-demographic variables. When examining the potential role of neighborhood factors, we found no significant differences by assets, perceptions of community-wide issues, nor neighborhood social cohesion.

### Independent adjusted effects of life course determinants on unhealthy days

Table 2 presents separate models for each determinant, adjusting by age, sex, race/ethnicity, education, income, community assets, community issues, and neighborhood cohesion. ACE was associated with 23 %-increased odds of poor HRQoL (≥14 unhealthy days) in adulthood (AOR = 1.23; 95 % CI: 1.06, 1.43). Increasing stress was also associated with increased likelihood of reporting ≥14 unhealthy days (AOR = 1.07; 95 % CI: 1.03, 1.10). Sleep disturbances were strongly associated with reports of 14 or more unhealthy days. Specifically, any additional sleep deprived day was associated with 9 times higher odds of reporting ≥14 unhealthy days (AOR = 8.86; 95 % CI: 3.61, 21.77). After adjusting for covariates, no associations were found for smoking, alcohol use, physical activity, fruits and vegetable intake, or social support. Based on these findings, we selected stress and sleep disturbance as potential mediators that needed to be examined in the test of mediation.

**Table 2:** Independent unadjusted and adjusted effects of adverse childhood experiences and current social determinants on the odds of reporting ≥14 days of unhealthy days per month

Variable^c^	≥14 days of unhealthy days per month	
	OR (95 % CI)	AOR^b^ (95 % CI)
Adverse childhood experiences	1.19 (1.05, 1.34)^a^	1.23 (1.06, 1.43)^a^
Stress	1.05 (1.03, 1.08)^a^	1.07 (1.03, 1.10)^a^
Social support	.99 (.98, 1.01)	.99 (.97, 1.01)
Smoking	1.50 (.79, 2.86)	1.30 (.59, 2.89)
Alcohol use	2.54 (1.02, 6.33)^a^	2.78 (.93, 8.31)
Physical activity	.90 (.46, 1.69)	.66 (.31, 1.39)
Fruits and vegetable intake	1.04 (.55, 1.96)	1.32 (.61, 2.83)
Sleep disturbances	6.08 (3.03, 12.24)^a^	8.86 (3.61, 21.77)^a^

^a^Statistically significant 95 % CI

^b^Separate models for each determinant, adjusting by age, sex, race/ethnicity, education, income, community assets, community issues, and neighborhood cohesion

^c^With the exception of smoking (yes/no), all other predictor variables were on a continuous measurement scale

### Test of mediation

Table 3 presents the stepwise assessment of controlled direct effects of ACE, stress, and sleep disturbances on HRQoL. In Model 1, it can be appreciated that ACE significantly predicted poor HRQoL. The model correctly predicted 73 % of cases with excessive unhealthy days. In Model 2, this relationship was explained by stress and the model had greater explanatory power (Pseudo-R^2^ = 0.32) than the first model. In Model 3, the addition of sleep disturbances improved the explanatory power (Pseudo-R^2^ = 0.44) and the percent of correct predictions was increased to 82.1 %. All three models demonstrated adequate goodness of fit (Hosmer-Lemeshow non-significant). On the other hand, these three initial logistic regression models only indicate measures of associations with controlled effects and cannot estimate the magnitude of mediated or indirect effects (i.e., effect of ACE on Stress, plus the effect of stress on unhealthy days) or if the mediation is partial or total [47].

**Table 3:** Excessive unhealthy (≥14) days predicted by adverse childhood experiences and step-wise inclusion of mediators

Variables	Model 1		Model 2		Model 3	
	B	(95 % CI)^a^	B	(95 % CI)^a^	B	(95 % CI)^a^
Main Predictor						
Adverse childhood experiences	.21^b^	(.05, .43)	.104	(−.06, .34)	.03	(−.24, .28)
Mediators						
Stress			.061^b^	(.03, .158)	.05^b^	(.01, .12)
Sleep disturbances					.10^b^	(.06, .23)
Confounders						
Age	.01	(−.027, .04)	.03	(−.01, .08)	.03	(−.01, .09)
Sex	−.19	(−1.33, .76)	−.19	(−1.67, .95)	.21	(−1.37, 1.84)
Education	.78	(−.36, 2.28)	1.35^b^	(.05, 3.48)	1.85^b^	(.40, 4.38)
Marital status	.78	(−.19, 2.40)	.55	(−.72, 2.02)	.87	(−.47, 3.06)
Non-Hispanic black	−.45	(−2.44, 1.65)	−.17	(−1.97, 1.85)	−.65	(−3.01, 1.41)
Hispanic or Latino	−.81	(−3.08, 1.40)	−.72	(−2.93, 1.65)	−.97	(−3.64, 1.24)
US$20,001–40,000	−.44	(−1.88, .71)	−.02	(−1.68, 1.34)	−.42	(−2.76, 1.20)
≥US$40,001	−.29	(−19.69, 1.30)	−.31	(−19.29 1.13)	−.30	(−20.16, 1.85)
Community assets	−.05	(−.19, .08)	.07	(−.08, .26)	.07	(−.11, .28)
Neighborhood issues	−.02	(−.23, .15)	−.12	(−.34, .08)	−.17	(−.49, .03)
Neighborhood cohesion	−.01	(−.03, .01)	−.01	(−.05, .02)	−.01	(−.05, .02)
Constant	−.76	(−5.04, 2.81)	−4.32^b^	(−16.16, −.47)	−4.7	(−13.87, −.05)
Model Chi-square [df]	22.48 [7]		38.32 [11]		56.26 [14]	
Nagelkerke R^2^	.18		.32		.44	
% Correct predictions	73.2		80.8		82.1	
Goodness of fit p-value	.44		.55		.38	

*Model 1:* binary logistic regression with Unhealthy Days as outcome, ACE as predictor, and controlling for age, gender, education, marital status, race/ethnicity, income, community assets, neighborhood issues, neighborhood cohesion as covariates

*Model 2:* Model 1, adding stress (mediator 1)

*Model 3:* Model 2, adding sleep disturbance (mediator 2)

*N* = 151 for model 1,2, and 3

^a^Bootstrapped 95 % confidence intervals based on 1000 bootstrapped samples. All values rounded to two digits.

^b^Indicates that the coefficient is statistically significant at, at least, the .05 level

Table 4 presents the decomposition of effects into total, indirect, and direct effects for the HRQoL measure. The three essential conditions to determine mediation were established, even after controlling for SES and neighborhood level factors. First, ACE (main predictor) independently predicted self-reports of ≥14 unhealthy days due to physical or mental illness (outcome). Second, ACE significantly predicted two mediators, namely perceived stress and sleepless days. Third, these mediators (stress and sleep disturbance) significantly predicted unhealthy days.

**Table 4:** Mediation of stress on the relationship between adverse childhood experiences and excessive unhealthy days

Path	Coefficients^a, b^	S.E.
IV to Mediators (a paths): ACE to Stress and Sleep		
Perceived stress	1.64^e^	.54
Sleep disturbance	1.09^e^	.30
Direct Effects of Mediators on DV (b paths)		
Perceived stress	.05^e^	.02
Sleep disturbance	.10^e^	.03
Total Effect of IV on DV (c path)		
Adverse childhood experiences	.19^e^	.07
Direct Effect of IV on DV (c’ path)		
Adverse childhood experiences	.03	.09
Partial Effect of Control Variables on DV		
Age	.03	.02
Sex	.18	.57
Education	1.85^e^	.63
Marital status	.79	.58
Race/ethnicity	−.43	.49
Household income	−.23	.39
Community assets	.07	.07
Neighborhood issues	−.16	.09
Neighborhood cohesion	−.01	.01
Indirect Effects of IV(ACE) on DV(Unhealthy days) through Proposed Mediators (ab paths)^c^		
	Data	Boot (95 % CI)^d^
Total	.19	.22 (.06, .33)
Perceived stress	.08	.09 (.01, .21)
Sleep Deprivation	.11	.13 (.03, .21)

^a^All values rounded to two digits. ^b^Linear regressions for ‘a paths’ for perceived stress and sleep deprivation. All other coefficients derived with binary logistic regression. ^c^Formulas: Total Effect: c = c’ + ab; amount of mediation or indirect effect: ab = c - c’. ^d^Based on 1000 bootstrapped samples. ^e^Path coefficient significant at *p* < 0.05

Mediated effects were significant for stress (β = 0.08; 95 % CI: 0.01, 0.21) and sleep disturbances (β = 0.11; 95 % CI: 0.03, 0.21) on the relationship between ACE and excessive unhealthy days. It can be noted that when testing for mediation the direct effect of ACE on unhealthy days (c’ path) became non-significant, which indicates total mediation. The joint total mediated effect of stress and sleep disturbances was 0.19 (95 % CI: 0.06, 0.33). By exponentiation of the later beta coefficient, we obtain an OR of 1.20 (95 % CI: 1.06, 1.39). In other words, there is an increase of 20 % in the odds of reporting ≥14 unhealthy days for every additional ACE, which is mediated by stress and sleep disturbances in the adulthood. It should be highlighted that such effect size is very close to the independent controlled effect of ACE on unhealthy days that was noted in Model 1 of Table 3.

## Discussion

We found that exposure to ACE is linked to impaired adult HRQoL, with mediation effects modulated by stress and sleep disturbances. An implied hypothesis of this study was that there was an association between ACE and HRQoL. Our study confirms this association and is consistent with previous findings [7, 11–15]. Unique to our study, however, is the way in which ACE was measured using the widely established theoretical framework, the LCP, to capture cumulative life experiences over time.

The LCP provided the theoretical guidance to frame the inquiry, while CBPR fostered active community engagement in the design of research questions that are relevant to the community context and in the collection of reliable experiential information [52, 53]. The multi-domain conceptualization and multi-level nature of LCP along with CBPR approaches have the potential of improving the assessment and deepening the understanding of determinants of health disparities. It is precisely because of these notable features, namely, integration (i.e., risk/protection), multilevel (i.e., individual, families, and communities), and time-orientation (i.e., life span), that the LCP was able to assist in explaining why and how health disparities occur.

Another hypothesis in this study was that certain symptoms are expressed later in life among victims of ACE, resulting in a symptomatology complex that includes a physically and emotionally poor quality of life. Our study is novel in this perspective because we conducted a detailed and robust mediation analysis, which mapped out mechanismal pathways that could potentially explain early life experiences and subsequent impaired HRQoL. Specifically, we observed that ACE victims in this study were more likely to have heightened stress levels and sleep disturbances, a finding that underscores the importance of their role as potential mediators linking ACE to HRQoL. There are clinical as well as public health implications of these observations. In particular, they represent potential avenues for intervention to minimize the negative impact of ACE on HRQoL.

There are two main approaches of effective strategies to improve the HRQoL among adults impacted by ACE. The first is to identify those impacted by ACE early in life and intervene immediately. However, there are circumstances (e.g., poverty, hidden abuse, etc.) which may elude early detection. In such instances, the other approach would be detection of ACE through screening measures with intervention coming later in life. For example, those impacted by ACE may exhibit somatization by expressing symptoms such as sleep and stress disturbances and may greatly benefit from improved tertiary care.

It is important to place our results within the context of a methodological limitation, namely that the survey upon which our findings were based was cross-sectional. Nonetheless, it is noteworthy that a merit of the study instrument is that the questions were framed within specific temporal windows, and therefore, the temporal relationships between ACE, HRQoL, and the other factors included could be established. Another shortcoming of our analysis is that while our assessment was based on a representative community sample, it cannot be taken as directly generalizable to the entire US population, due to the selective context of our project (e.g., mostly low income, African American women). The advantage of this limitation, however, is that the study addressed an important cause of low HRQoL in a socio-economically disadvantaged setting that stands to benefit most from appropriate and targeted interventions for victims of ACE.

## Conclusions

In summary, our findings indicate that adversity in childhood continues to affect the mental and behavioral health trajectory of adults. Thus, we recommend the implementation of community health programs aimed at improving psychological well-being by reducing high stress levels, particularly among individuals who have suffered childhood trauma. We suggest an approach from the “womb to the tomb,” which starts by addressing psychological well-being and behavioral health of expecting mothers and continue to provide support to minimize the effect of unresolved childhood traumas, as well as the linkages to professional and community supports throughout the life span. This offers a potential path to the improvement of HRQoL for victims impacted by ACE.

## Additional file

Additional file 1:
**Survey Questions and Flow.** (PDF 130 kb)

## Abbreviations

ACE: Adverse childhood experiences
HRQoL: Health-related quality of life
SD: Standard deviation
AOR: Adjusted odds ratio
CI: Confidence interval
CDC: Centers for Disease Control and Prevention
CBPR: Community-based participatory research
USF: University of South Florida
CAB: Community Advisory Board
LCP: Life Course Perspective
BRFSS: Behavioral Risk Factor Surveillance System
