# Supplementary material for: Adverse childhood experiences and health-related quality of life in adulthood: revelations from a community needs assessment
Source: Health Qual Life Outcomes. 2015 Aug 11;13:123. doi: 10.1186/s12955-015-0323-4 (PMC4530491; doi:10.1186/s12955-015-0323-4)
Supplement: Additional file 1: — Survey Questions and Flow. (PDF 130 kb) [file 12955_2015_323_MOESM1_ESM.pdf]

droidSURVEY

**Survey Name:**	Copy of ENGLISH_Your Health Throughout the Life Course
**Description:**	Project Survey
**Introduction:**	Welcome to the survey. Touch the Start button to continue
**Conclusion:**	Thank you for completing the survey

Survey Outline Only - Actual Survey Outlook on Tablets Screen Differs from Actual Display

Survey Questions and Flow

	Question Type	Question	Settings	Statements	Answers/Ratings	Next Question
1 :	Information	SURVEY INSTRUCTIONS: This survey should only take about 20-30 minutes of your time. Your answers will be completely anonymous and confidential. Touch on the screen to select your answer. Click the NEXT button to continue. Click the BACK button to return.				2
2 :	Single Select	How long have you lived in this neighborhood? (in years)	◦ Answer Required			3
					Less than 1 year	3
					1 to 5 years	3
					6 to 10 years	3
					11 to 20 years	3
					More than 20 years	3
3 :	Single Select	Please select your residential zip code:	◦ Answer Required			4
					33602	4
					33603	4
					33605	4
					33607	4
					33610	4
4 :	Single Select	Are you Male or Female?	◦ Answer Required			5
					Male	5
					Female	5
5 :	Numeric	How old are you? (in years completed)	◦ Answer Required			6
6 :	Single Select	How do you feel now about using this tablet device to answer the survey?	◦ Answer Required			7
					I like to take the survey using this device.	7
					I'd prefer my survey in paper (ask the research staff for help)	7
7 :	Image	SECTION 1 OF 7: LIFE IN THE NEIGHBORHOOD				8
8 :	Multi Select	Which of the following resources are available to you in your community? MARK ALL THAT APPLY. Scroll down to find more choices.	◦ Specify Answer			9

				Churches<br>Colleges, universities<br>Police/Fire Department<br>Hospitals and clinics<br>Pharmacies<br>Mental health facilities<br>Libraries<br>Schools<br>Public transportation<br>Parks and recreational facilities<br>Local businesses<br>Community centers, such as YMCA or Family Centers<br>Cultural activities, museums, or musical forums<br>NONE of the resources listed<br>Other, please indicate	
**9 :**	Multi Select	Which of the following is a problem in the neighborhood? Scroll down to find more choices.	◦ Specify Answer	Abandoned houses, factories, or buildings<br>Vandalism, high crime rate<br>Homeless persons/panhandling<br>Drug/alcohol-related problems<br>Trash or litter in empty lots, streets, or properties<br>Poor quality of schools, or libraries<br>Poor quality grocery<br>Lack of affordable shopping<br>Poor police response<br>Lack of quality of recreation, parks, or sports facilities<br>Junkyards, gasoline stations, and other non-residential<br>Dogs and uncontrolled animals<br>Industrial hazards such as incinerators, chemical plants, and hazardous waste sites<br>NONE of the problems listed<br>Other, please indicate	10
**10 :**	Image	SECTION 2 OF 7: SOCIAL CONNECTIONS			11
**11 :**	Numeric	About how many close friends and close relatives do you have (people you feel at ease with and can talk to about what is on your mind)? (SUM ALL FRIENDS AND RELATIVES)	◦ Answer Required		12
			◦ People around here are willing to help their neighbors<br>◦ This is a close-knit		

**12 :**	Grid Scale	In thinking about the people in the neighborhood in which you live now, how strongly do you agree or disagree that:	◦ Answer Required	neightborhood<br>◦ People in this neighborhood generally get along with each other<br>◦ People in this neighborhood share the same values		13
					Strongly Disagree<br>Disagree<br>Neither Agree nor Disagree<br>Agree<br>Strongly Agree	
**13 :**	Grid - Single Select	How often is each of the following kinds of support available to you if you need it?	◦ Answer Required	◦ Someone to confide in or talk about yourself or your problems<br>◦ Someone to share your most private worries and fears with<br>◦ Someone to help you if you were confined to bed<br>◦ Someone to prepare your meals if you were unable to do it yourself<br>◦ Someone to get together with for relaxation		14
					None of the time<br>A little of the time<br>Some of the time<br>Most of the time<br>All of the time	
**14 :**	Image	SECTION 3 OF 7: HEALTH AND QUALITY OF LIFE				15
**15 :**	Single Select	Would you say that in general your health is:	◦ Answer Required			16
					Excellent	16
					Very Good	16
					Good	16
					Fair	16
					Poor	16
					Don't know/not sure	16
			◦ Answer			

16:	Numeric	Now thinking about your physical health, which includes physical illness and injury, for how many days during the past 30 days was your physical health not good? (If none, put 0 for zero days)	Required<br>◦ Prefix-Days		17
17:	Numeric	Now thinking about your mental health, which includes stress, depression, and problems with emotions, for how many days during the past 30 days was your mental health not good? (If none, put 0)	◦ Answer Required<br>◦ Prefix-Days		18
18:	Numeric	During the past 30 days, for about how many days did poor physical or mental health keep you from doing your usual activities, such as taking care of yourself, work, or recreation? (if none, put 0)	◦ Answer Required<br>◦ Prefix-Days		19
19:	Multi Select	In the LAST 12 MONTHS, have you suffered any of the following health problems? Scroll down for more choices.	◦ Answer Required<br>◦ Specify Answer<br>◦ Min Answers-1		20
				Arthritis/rheumatism	
				Back or neck problem	
				Walking problem	
				Fractures, or bone/joint injury	
				Infections	
				Lung/breathing problem	
				Hearing problem	
				Eye/vision problem	
				Heart problem	
				Stroke problem	
				Hypertension/high blood pressure	
				Diabetes or high blood sugar	
				Stress	
				Cancer	
				Emotional problems/Depression/Anxiety	
				Drinking problem/Alcoholism	
				Substance abuse	
				Overweight or obesity	
				NONE of these problems	
				Other impairment or health problem	
20:	Numeric	During the past 30 days, for about how many days have you felt you did NOT get ENOUGH REST or SLEEP?	◦ Answer Required<br>◦ Prefix-Days		21
21:	Single Select	In a scale from 1 to 10, where do you think you stand at this time in your life, relative to other people in your community?	◦ Answer Required		22
				1 - WORST OFF	22
				2	22
				3	22
				4	22

					5	22
					6	22
					7	22
					8	22
					9	22
					10 - BEST OFF	22
**22 :**	Image	SECTION 4 OF 7: STRESS AND UNFAIR TREATMENT				23
**23 :**	Grid - Single Select	In the LAST MONTH, how often have you...	◦ Answer Required	◦ ...felt that you were unable to control the important things in your life?<br>◦ ...felt confident about your ability to handle your personal problems?<br>◦ ...felt that things were going your way?<br>◦ ...felt difficulties were piling up so high that you could not overcome them?		24
					Never	
					Almost Never	
					Sometimes	
					Fairly Often	
					Very Often	
**24 :**	Multi Select	In the LAST 12 MONTHS, what things have you felt most stress about? (MARK ALL THAT APPLY)	◦ Answer Required<br>◦ Min Answers-1			25
					Family	
					Money	
					Housing	
					Children	
					Work	
					Food	
					Partner	
					Other	
					Not applicable/No stress in last 12 months	
				◦ I talk to trusted friends or counsel family members		

**25:**	Grid - Single Select	When thinking about solving my difficulties or problems...	- Answer Required	- The spiritual support/faith in God received is my comfort and strength to guide me<br>- I wait for the spirit/God to take control and work it out<br>- I try to come up with possible solutions on my own		26
					Never<br>Almost Never<br>Sometimes<br>Fairly Often<br>Very Often	
**26:**	Multi Select	When you have problems, where do you go for support or assistance: (MARK ALL THAT APPLY)	- Answer Required<br>- Specify Answer<br>- Min Answers-1			27
					Friends/Neighbors<br>Public programs<br>Non-profit programs<br>Church or faith-based service<br>Family<br>None of the above<br>Other	
**27:**	Grid - Single Select	Have you experienced discrimination, hassled, or made inferior because of your personal characteristics?	- Answer Required	- At school<br>- Getting hired or getting a job<br>- At work<br>- Getting housing<br>- Getting medical care<br>- Getting service in a store or restaurant<br>- Getting credit, bank loans, or a mortgage<br>- On the street or in a public setting<br>- From the police or in the courts		28
					NO, NEVER<br>At least once	

				2-3 times	
				4 or more times	
**28 :**	Multi Select	What do you think was/were the reason(s) for that experience? (ALL THAT APPLY)	- Answer Required<br>- Specify Answer<br>- Min Answers- 1		29
				Your ancestry or national origins	
				Your gender	
				Your race	
				Your ethnicity	
				Your age	
				Your religion	
				Your height or weight	
				Your shade of skin color	
				Your sexual orientation	
				Your education or income level	
				A physical disability	
				Your personal beliefs	
				Other	
**29 :**	Information	SECTION 5 OF 7: LIFE STYLE QUESTIONS			30
**30 :**	Single Select	Have you smoked at least 100 cigarettes in your entire life?	- Answer Required		31
				Yes	31
				No	31
				Don't know / Refuse to answer	31
**31 :**	Single Select	During your first 18 years of life did anyone in your household smoke?	- Answer Required		32
				Yes	32
				No	32
				Don't know	32
**32 :**	Single Select	How often do you drink alcoholic beverages?	- Answer Required		33
				I do not drink at this time/I stopped drinking for more than a year now	33
				Less than Once a Month	33
				Once a Month	33
				2-3 Times a Month	33
				Once a Week	33
				2-3 Times a Week	33
				4-6 Times a Week	33
				Everyday	33

					Refuse to answer/Not sure	33
33:	Single Select	Do you use sometimes use drugs for recreation, or not prescribed by your doctor?	Answer Required			34
					I do not use drugs/I stopped using drugs for more than 1 year now	34
					Less than Once a Month	34
					Once a Month	34
					2-3 Times a Month	34
					Once a Week	34
					2-3 Times a Week	34
					4-6 Times a Week	34
					Everyday	34
					Refuse to answer/Not sure	34
34:	Grid - Single Select	In a typical month, how often do you eat the following types of food?	Answer Required	Bread, cereal, rice, and pasta<br>Fruits and vegetables<br>Dairy products (milk, yogurt, and cheese)<br>Meat, poultry, or pork<br>Seafood (fish, shrimp, tuna, etc)<br>Fried food (High-fat foods)<br>Salty foods or snacks (like salted crackers or pretzels.)<br>Sugary foods (sweets or pastries)<br>Coffee, sodas, or energy drinks		35
					Once a Month or less	
					2-3 Times a Month	
					Once a Week	
					2-3 Times a Week	
					4-6 Times a Week or more	
35:	Single Select	During the past month, about how many days per week did you exercise for recreation or to keep in shape (activities that make you sweat)?	Answer Required			36
					Once a Month or less	36
					2-3 Times a Month	36
					Once a Week	36
					2-3 Times a Week	36
					4-6 Times a Week or more	36
36:	Information	The next questions are about any personal concerns you may have about AIDS...				37

**37 :**	Single Select	Are you currently sexually active with a partner?	◦ Answer Required		38
				Yes	38
				No	38
				Refuse to answer/Don't know	38
**38 :**	Single Select	In the next 5 years, is it likely or unlikely that the average [man/woman] in your age group in your community will get the AIDS/HIV virus?	◦ Answer Required		39
				Very likely	39
				Somewhat likely	39
				Somewhat unlikely	39
				Very unlikely	39
				Don't know/Refuse to Answer	39
**39 :**	Image	SECTION 6 OF 7: FAMILY HISTORY AND CHILDHOOD			40
**40 :**	Grid - Single Select	When you were growing up, how often did the following situations happen in your family?	◦ Answer Required	◦ We talked about our problems.<br>◦ When we argued, we listened to "both sides of the story."<br>◦ We took time to listen to each other.<br>◦ We pulled together when things were stressful.<br>◦ We were able to solve our problems.	41
				Never	
				Rarely	
				Sometimes	
				Often	
				All of the time	
**41 :**	Grid - Single Select	During your first 18 years of life, how often the following types of supports were available to you?	◦ Answer Required	◦ I had others who would listen when I needed to talk about my problems.<br>◦ When I was lonely, there were several people I can talk to.<br>◦ I had someone to turn to if I needed food or housing.<br>◦ If there was a	42

			crisis, I had others I can talk to.<br>◦ If I needed help in school, I would know where to go for help.		
				Never	
				Rarely	
				Sometimes	
				Often	
				All of the time	
**42:**	Information	The following questions ask about difficult experiences when you were growing up. All information is anonymous and confidential. Your responses will help us understand how much need exists in the community for addressing sad or traumatic experiences among children.			43
**43:**	Single Select	Did a parent or other adult in the household often or very often swear at you, insult you, put you down, or humiliate you? OR Act in a way that made you afraid that you might be physically hurt?	◦ Answer Required		44
				Yes	44
				No	44
				Refuse to answer/Don't know	44
**44:**	Single Select	Did a parent or other adult in the household often or very often...push, grab, slap, or throw something at you? OR Ever hit you so hard that you had marks or were injured?	◦ Answer Required		45
				Yes	45
				No	45
				Refuse to answer/Don't know	45
**45:**	Single Select	Did an adult or person at least 5 years older than you ever..touch your in a sexual manner or fondle you or have you touch their body in a sexual way? OR Attempt or actually have intercourse with you?	◦ Answer Required		46
				Yes	46
				No	46
				Refuse to answer/Don't know	46
**46:**	Single Select	Did you often or very often feel that...No one in your family loved you or thought you were important or special? OR Your family didn't look out for each other, feel close to each other, or support each other?	◦ Answer Required		47
				Yes	47
				No	47
				Refuse to answer/Don't know	47
**47:**	Single Select	Did you often or very often feel that...You didn't have enough to eat, had to wear dirty clothes, and had no one to protect you?	◦ Answer Required		48
				Yes	48
				No	48
				Refuse to answer/Don't know	48
**48:**	Single Select	Were your parents ever separated or divorced?	◦ Answer Required		49
				Yes	49
				No	49

				My parents were never married	49
				Refuse to answer/Don't know	49
**49:**	Single Select	Was your female caretaker: Often or very often pushed, grabbed, slapped, or had something thrown at her? OR Sometimes, often, or very often kicked, bitten, hit with a fist, or hit with something hard? OR Ever repeatedly hit at least a few minutes?	◦ Answer Required		50
				Yes	50
				No	50
				Refuse to answer/Don't know	50
**50:**	Single Select	Did you live with anyone who was a problem drinker or alcoholic or who used street drugs?	◦ Answer Required		51
				Yes	51
				No	51
				Refuse to answer/Don't know	51
**51:**	Single Select	Was a household member depressed or mentally ill, or did a household member attempt suicide?	◦ Answer Required		52
				Yes	52
				No	52
				Refuse to answer/Don't know	52
**52:**	Single Select	Did a household member go to prison?			53
				Yes	53
				No	53
				Refuse to answer/Don't know	53
**53:**	Image	SECTION 7 OF 7: SOCIODEMOGRAPHICS			54
**54:**	Single Select	What is the highest level of education you have completed?	◦ Answer Required		55
				No schooling completed	55
				Nursery school to 8th grade	55
				Some high school, but not currently studying	55
				Currently in high school or working toward high school or GED diploma	55
				High school graduate, diploma or the equivalent (GED)	55
				Trade/technical/vocational training	55
				Some college credit, no degree	55
				Associate degree	55
				Bachelor's Degree	55
				Master's Degree or Professional or Doctoral Degree	55
**55:**	Single	Please indicate your current marital status?	◦ Answer		56

	Select		Required		
				Single, never married	56
				Married or domestic partnership	56
				Widowed	56
				Divorced	56
				Separated	56
				Other	56
				Refuse to Answer	56
**56:**	Single Select	Are you of Latino, Hispanic, or Spanish descent?	◦ Answer Required		57
				No	57
				Yes	57
				Refuse to answer/Don't know	57
**57:**	Multi Select	Which racial group do you identify yourself with more? (One or more if applicable)	◦ Answer Required<br>◦ Min Answers-1		58
				Asian	
				Black or African American	
				White or Caucasian	
				American Indian or Alaska Native	
				Pacific Islander or Native Hawaiian	
				Other race	
				Don't know/Refuse to answer	
**58:**	Single Select	Which of the following best describes your employment status?	◦ Answer Required		59
				Employed for wages	59
				Self-employed	59
				Out of work and looking for work	59
				Out of work and not currently looking for work	59
				A homemaker	59
				A student	59
				Military	59
				Retired	59
				Unable to work	59
				Refuse to answer/Don't know	59
**59:**	Multi Select	Do you receive any of the following assitance? (MARK ALL THAT APPLY)	◦ Answer Required<br>◦ Min Answers-1		60
				Section 8 (Housing Choice Voucher Program)	
				Food Stamps (SNAP)	
				School free or reduced lunches	

					Medicaid	
					SSI (Supplemental Security Income)	
					TANF (Temporary Assistance for Needy Families, also known as "welfare")	
					Other assistance	
					Refuse to answer/Don't know	
**60:**	Single Select	To the best of your knowledge, what is your household annual income (in US dollars)? REMEMBER: Your information is confidential and anonymous	◦ Answer Required			61
					$0-10,000	61
					$10,001 - 20,000	61
					$20,001 - 30,000	61
					$30,001 - 40,000	61
					$40,001 - 50,000	61
					More than $50,000	61
					Don't know / Refuse to answer	61
**61:**	Numeric	How many people are currently living in your household, including yourself, that live on that income?	◦ Prefix- Number<br>◦ Suffix- people			62
**62:**	Grid - Single Select	Thanks for making it to this point, before you go, please tell us how you felt about taking questions with this portable device	◦ Answer Required	◦ I think that I would like to use this survey device frequently<br>◦ I found the survey device unnecessarily complex<br>◦ I thought the survey device was easy to use<br>◦ I thought there was too much inconsistency in the survey<br>◦ I would imagine that most people would learn to use this survey system very quickly<br>◦ I found the survey device was cumbersome to use<br>◦ I felt very confident using this system<br>◦ I needed to learn a lot of things before I could get going with the		63

			survey		
				Strongly Disagree	
				Disagree	
				Neutral	
				Agree	
				Strongly Agree	
**63:**	Single Select	Before you took this survey, what was your previous experience with tablet computers or portable devices like this?	○ Answer Required		64
				This is the first time I use a device like this	64
				I have used a few times tablet PCs, smartphones, or i-phones	64
				I use devices like this regularly	64
**64:**	Information	THANK YOU FOR YOUR RESPONSES Click NEXT TO SUBMIT your responses. If you have questions about this survey, please tell the research staff, or call REACHUP Inc at: (813) 712-6300 You can also email at: cbprtampa@gmail.com			Conclusion
